# Mitochondrial Sequence Variation, Haplotype Diversity, and Relationships Among Dromedary Camel-Types

**DOI:** 10.3389/fgene.2021.723964

**Published:** 2021-08-30

**Authors:** Randa Alaqeely, Bader H. Alhajeri, Faisal Almathen, Hasan Alhaddad

**Affiliations:** ^1^Department of Biological Sciences, Kuwait University, Kuwait City, Kuwait; ^2^Department of Veterinary Public Health, College of Veterinary Medicine, King Faisal University, Al-Ahsa, Saudi Arabia; ^3^The Camel Research Center, King Faisal University, Al-Ahsa, Saudi Arabia

**Keywords:** camel, mtDNA, haplogroup, polymorphism, population

## Abstract

Dromedary camels are outstanding livestock that developed efficient abilities to tolerate desert conditions. Many dromedary camel-types (i.e., named populations) exist but lack defined specific breed standards, registries, and breeders’ governing organizations. The breed status of dromedary camel-types can partly be assessed by exploring mitochondrial DNA (mtDNA) variation. Accordingly, this study aimed to examine the breed status and the inter-population relationships of dromedary camel-types by analyzing sequence variation in the mtDNA control region and in three coding genes [*cytochrome b*, *threonine, and proline tRNA*, and part of the displacement loop (D-loop)] (867 bp region). Tail hair samples (*n* = 119) that represent six camel-types from Kuwait were collected, extracted, sequenced, and compared to other publicly available sequences (*n* = 853). Within the sequenced mitochondrial region, 48 polymorphic sites were identified that contributed to 82 unique haplotypes across 37 camel-types. Haplotype names and identities were updated to avoid previous discrepancies. When all sequences were combined (*n* = 972), a nucleotide diversity of 0.0026 and a haplotype diversity of 0.725 was observed across the dromedary-types. Two major haplogroups (A and B) were identified and the B1 haplotype was predominant and found in almost all dromedary-types whereas the A haplotypes were more abundant in African regions. Non-metric multidimensional scaling revealed an increased similarity among Arabian Peninsula “Mezayen” camel-types, despite their defining coat colors. The relationships among dromedary camel-types can partly be explained by mtDNA. Future work aimed at a deeper understanding of camel-type breed status should focus on a high number of nuclear markers.

## Introduction

The dromedary, *Camelus dromedarius*, is well-known for its adaptations to harsh desert conditions. The adaptations include structural (Alsafy et al., 2013; Achaaban et al., 2016), physiological (Adamsons et al., 1956; Schmidt-Nielsen et al., 1956), and behavioral traits (Mitchell et al., 2002; Djazouli Alim et al., 2012). The natural adaptations of the dromedaries were anthropologically exploited via (1) its domestication around 2000–3000 years B.C., (2) the expansion of their uses, and (3) the development of unique populations (i.e., camel-types) (Uerpmann and Uerpmann, 2002; Almathen et al., 2016; Orlando, 2016). However, unlike other domesticated animals (e.g., cattle, sheep, horses, dogs, and cats), dromedaries do not currently have breed definitions, standards, registries, or breeders’ organizations (Arman, 2007; Lynghaug, 2009; Alhaddad and Alhajeri, 2019). Named dromedary populations are locally known and occasionally documented, but little is known about their breed status. As a result, named camel populations are referred to here as “camel-types” instead of breeds (Alaskar et al., 2021). Porter et al. (2016) has reported about 200 different camel-types, yet many displayed overlapping characteristics and thus may include types with synonymous names. Using a few STR markers, AlAskar et al. (2020) partially explored the breed status of dromedary camel-types; the inconclusive conclusions suggested exploring the populations using mitochondrial DNA (mtDNA) sequence variation or a higher number of nuclear markers.

Mitochondrial DNA variation can be used to gain a better understanding of dromedary populations, types, evolution, and domestication history. Analyses of mtDNA variation have been used to identify (1) maternal lineages (Jansen et al., 2002; Yang et al., 2018), (2) wild ancestry (Kadwell et al., 2001; Naderi et al., 2008; Kimura et al., 2010), and (3) geographic origins of different species (Cieslak et al., 2010; Di Lorenzo et al., 2015; Almathen et al., 2016). With a focus on domesticated animals, mtDNA variation have been used to study the breed relationships of Bactrian camels (Ming et al., 2017), horses (Hristov et al., 2017), donkeys (Cozzi et al., 2018), goats (Kibegwa et al., 2016), and cattle (Di Lorenzo et al., 2018). The mitochondrial genome of dromedary camels is ∼16.6 kb in length and consists of genes encoding *tRNAs* (22 genes), *rRNAs* (2 genes), *sequence tagged sites* (*STS*) (3 sites), *NADH dehydrogenase* (7 genes), *cytochrome c oxidase* (3 genes), *ATP synthase* (2 genes), and *cytochrome b* (a single gene) (GenBank accession number: NC_009849, Huang et al., unpublished). The control region is the longest within the mitochondrial genome (1,124 bp) and the most variable non-coding region (Stoneking et al., 1991). The displacement loop (D-loop), which is located within the control region, exhibits the highest levels of polymorphism and accordingly is used for evolutionary studies (McMillan and Palumbi, 1997). mtDNA variation has been investigated in dromedary camels with a focus on the D-loop in addition to *tRNA* (mostly proline and threonine) and *cytochrome b* sequences. Using this localized sequence variation, Almathen et al. (2016) found that dromedary populations, combined based on country of origin, exhibited no clear phylogeographic clustering. Also, the authors reported two major haplogroups (A and B), which consisted of 76 haplotypes of different frequencies (Almathen et al., 2016). Nonetheless, the naming and assignment of haplotypes into haplogroups lacked a clear methodology, and the haplotype sequences and positions of mutation(s) were not reported.

The objectives of this study were to: (1) re-examine the molecular variation within an mtDNA region using 972 dromedary samples, (2) evaluate the molecular variation within and among dromedary camel-types, (3) classify and report haplogroups and haplotypes, (4) investigate the relationship between the identified haplotypes and the camel-type’s naming system and geography, (5) test the hypothesis that having an apparent selection criteria in specific camel-types may affect haplotype variability, and (6) evaluate the relationships within “Mezayen” camel-types also known as “beauty pageant camels.”

## Materials and Methods

### Samples and DNA Extraction

Tail-hair specimens (*n* = 119) of unrelated dromedary camels were selected from the Cdrom Archive (Alhaddad and Alhajeri, 2018, 2019) for the current study. Relatedness was avoided not only by looking at the information associated with each dromedary camel and its pedigree, but also by avoiding the inclusion of more than one sample per breeder when possible. Selected samples belonged to six dromedary camel-types: Majaheem, Sofor, Shaele, Shageh, Homor, and Waddah (Porter et al., 2016; Supplementary Table 1). DNA was extracted from approximately 30 tail-hair follicles using a DNA extraction kit (PureLink Genomic DNA Mini Kit, Thermo Fisher Scientific) following an established protocol (Alhaddad et al., 2019). The quality of the extracted DNA was evaluated using a 1.5% agarose gel and the quantity and purity of the extracted DNA was assessed using eight channel nanodrop spectrophotometry (NanoDrop^TM^ 8000 Spectrophotometer, Thermo Fisher Scientific^TM^) at the Biotechnology Center at Kuwait University.

### Amplifying and Sequencing of the Target Region Using PCR

A mtDNA fragment of 867 bp length was amplified using a primer-pair previously designed and published Almathen et al. (2016): CB_F 5′ CCTAGCATTTATCCCCGCACTA3′ and tPRO_R 5′ GGTTGTATGATGCGGGTAAATG 3′. This fragment included the end of *cytochrome b* (184 bp), transfer RNA *threonine and proline* (134 bp), and the beginning of a control region spanning STRs (549 bp). PCR reaction was carried out in a total volume of 20 μ*l* containing: 4–84 ng genomic DNA, 0.6 μM of each primer, 10 μ*l* of *Taq* PCR Master Mix Kit (Qiagen^TM^) and completed to the final volume with nuclease free water. The PCR cycle was as follows: (1) an initial denaturation step at 94°C for 3 min, (2) 40 cycles each of denaturation at 94°C for 30 s, annealing at 60°C for 45 s, extension at 72°C for 90 s, and (3) a final extension step at 72°C for 5 min. The amplified DNA product was visualized in a 1.5% agarose gel then purified using ExoSAP-IT^TM^ PCR Product Cleanup Reagent (Applied Biosystems^TM^) as recommended by the manufacturer.

Using both forward and reverse PCR primers, independently, the PCR product was sequenced using Sanger sequencing (BigDye Terminator v3.1 Cycle Sequencing Kit, Thermo Fisher Scientific) following the manufacturer’s protocol. The sequencing reaction was carried out in a final volume of 20 μ*l* containing: (1) 8 μ*l* of BigDye Terminator Master Mix, (2) 2 μ*l* of each primer (0.6 μM) in two separate reactions, (3) 8 μ*l* of deionized water, and (4) 2 μ*l* of the amplified PCR product. Sequencing products were purified using the BigDye XTerminator^TM^ Purification Kit (Applied Biosystems^TM^) and its protocol. Sequences were analyzed using ABI 3130XL Genetic Analyzer at the Biotechnology Center in Kuwait University and submitted to GenBank (accession numbers MT164347 – MT164465).

### Sequence Quality and Alignment

Each sequence was visually inspected for quality, and only sequences with a clear chromatogram were included in the downstream analyses. Sequences of each individual (one using the forward primer and one using the reverse primer) were subjected to manual editing and cleaning using FinchTV (FinchTV^®^ 1.5.0, Geospiza, Inc., Seattle, WA, United States). Cleaned forward and reverse sequences were aligned to obtain the consensus sequences using BioEdit v.7.2.5 (Hall, 2013). A multiple sequence alignment was created for the generated consensus sequences of all samples using the CLUSTALW method as implemented in MEGAX (Kumar et al., 2018). Although the flanking regions were obtained using designed primers, sequences were cropped to match/align them with publicly available sequences of previously established studies (Almathen et al., 2016).

Beside the generated sequences in this study, publicly available sequences (*n* = 759) were retrieved and used in this study (Accession numbers JX946206-JX946273 and KF719283-KF719290) (Almathen et al., 2016) in addition to 95 unpublished sequences obtained from Saudi Arabia (Almathen, unpublished). The combined sequences belonged to 37 dromedary camel-types from 21 countries (Supplementary Figure 1 and Supplementary Table 2).

### Nucleotide and Haplotype Diversities

Sequences were assigned into mitochondrial haplotypes (mitotypes) using DnaSP software v 6.0, (Rozas et al., 2017). Using *Pegas* package (Paradis, 2010) in R software (R Development Core Team, 2018), the nucleotide diversity (Nei, 1987) was calculated using the function (*nuc.div*) and the haplotype diversity using (*hap.div*). To compare the effect of using D-loop independently to the use of the D-loop in addition to coding genes in terms of mitochondrial haplotype assignment, two sequence sets were created. The first included the whole 867 bp mtDNA (including *cytochrome b*, *threonine, and proline tRNA*, and D-loop) for all sequences. The second set of sequences included only the control (D-loop) region (552 bp in length).

Haplotype frequencies were analyzed in relation to geography and the dromedary camel-type naming system. Mitochondrial haplotypes with a frequency less than 0.019 were considered as “low frequency.” The aforementioned cut-off was calculated using the sample size formula ($s\,s=\frac{Z^{2}\times p\times \left(1-p\right)}{c^{2}}$), where: *Z* = *Z* value (e.g., 1.96 for 95% confidence level), p, population proportion expressed as a decimal (0.5 was used), c, confidence interval, expressed as a decimal (e.g., 0.04 = ± 4). So, when applying a confidence level of 95%, confidence interval of 20, and population size equals the total obtained haplotypes of 82, it was found that the minimum required sample size for a haplotype to be 19 individuals.

### Amendment of the Existing Haplotypes Nomenclature

The initial haplogroup naming system (A and B) was retained as previously reported (Almathen et al., 2016) to avoid confusion. The major haplotypes extending from haplogroups A and B were renamed based on sequence similarity to the major groups and their frequencies. For example, five major haplotypes were identified in haplogroup B, with frequencies (in percentage) equal to 50.5, 7.7, 7.1, 6.8, and 2.9, which were named as B1, B2, B3, B4, and B5, respectively. Haplotypes with frequencies less than 1.9% (this cut-off is based on the sample size formula – see above) were named sequentially following the names of the major haplotypes. For instance, haplotypes that directly originated from B1 were given names sequentially from B6 to B29, and haplotypes that originated from B2 were named starting with B30. A total of 82 haplotypes were assigned to the two haplogroups, where haplogroup A had 24, and haplogroup B had 58 haplotypes.

### Mitochondrial DNA Relationships

Phylogenetic relationships between identified haplotypes were inferred using the Bayesian method, as implemented in MRBAYES software v 3.2.7a (Huelsenbeck and Ronquist, 2001; Ronquist et al., 2012). HKY + I + G (Hasegawa et al., 1985) nucleotide substitution model with gamma correction (α = 0.0221) was used as the best fitting model for the 82 identified haplotypes based on lowest Akaike Information Criterion with correction for small sample size (AICc) (Akaike, 1974) value using jModelTest v 2.1.10 (Posada, 2008). Two independent Markov Chain Monte Carlo runs of two million generations each were used, with trees sampled every 1000 generations from the posterior distribution; the first 25% of the trees were discarded as burn-in generations (Almathen et al., 2016). Clade support was determined using Bayesian posterior probabilities and the same starting tree was used for each chain. Nonetheless, using different random starting trees showed similar results. The convergence and stationary of the post-burn-in tress was confirmed as well as the post-burn-in effective samples size (ESS) using TRACER v 1.7.1 (Rambaut et al., 2018). The relationships between the identified haplotypes were summarized via Median Joining Networks (MJ) using NETWORK software v 5.0.1.1 (Bandelt et al., 1999). The generated networks were modified using Network Publisher v 2.1.2.5.

The level of genetic differentiation between the dromedary camel-types was deduced by performing an Analysis of Molecular Variance (AMOVA) and by calculating mtDNA pairwise genetic differences *F*_st_ using ARLEQUIN v 3.5.2.2 (Excoffier and Lischer, 2010). Pairwise genetic distances between samples were visually inspected in a non-metric multidimensional scaling (NMDS) plot using the R package *vegan* (Oksanen et al., 2013).

Dromedary camel haplotypes were compared to five ancient dromedary camel samples (KT334309-KT334313), four Bactrian camel samples (KF640731, FJ792680, FJ792683, and KF640727), four wild Bactrian camel samples (FJ792685, FJ792684, EF212038, and NC_009629), seven guanaco samples (JQ754689-JQ754692, JQ754705, AY535173, and AY535174), sixteen vicuña samples (JQ754672-JQ754688), and six horse samples (MH032886-MH032891). The relationship between dromedary camel haplotypes and other species was investigated via a neighbor joining tree with a bootstrap value based on 1000 iterations using MEGAX software (Kumar et al., 2018).

## Results

### Mitochondrial DNA Polymorphism and Nucleotide Diversity

**FIGURE 1:**
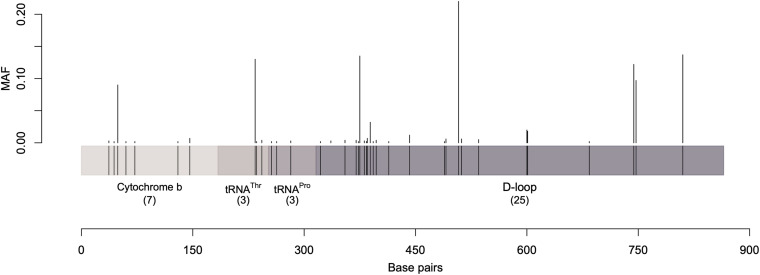
A graphical representation of the sequenced mtDNA fragment along with the parsimony informative sites and their minor allele frequency. Vertical lines represent the minor allele frequencies (MAF) of parsimony informative sites. Singletons were excluded.

Over the 867 bp sequence alignment, 48 polymorphic sites (substitutions) were observed, 38 of which were parsimony informative sites (i.e., contain at least two different nucleotides and at least two of them occur with a minimum frequency of two) while the rest were singleton variable sites (i.e., contain at least two different nucleotides with one of polymorphisms in overall high frequency). The majority of the detected polymorphisms were in the D-loop region (Figure 1 and Supplementary Tables 3, 4). The identified polymorphic sites resulted in 82 unique haplotypes. Analysis of the polymorphic sites at *cytochrome b* and *threonine tRNA* genes with MAF (minor allele frequency) greater than 0.1 showed no effect of geographic separation among samples (i.e., alleles are equally present in different dromedary camel-types of different countries as well as different continents).

The average nucleotide and haplotype diversities across all studied dromedary samples were 0.0026 and 0.725, respectively. The level of nucleotide diversity ranged from 0.0005 in the Targui camel-type to 0.0054 in the Hawari camel-type, and the level of haplotype diversity ranged from 0.378 in the Shageh dromedary camel-type to 0.964 in the Omani camel-type. Kuwaiti Majaheem samples displayed signs of homogeneity, having the smallest nucleotide diversity value within the type, and a low haplotype diversity. In fact, all camel-types from Kuwait (Majaheem, Sofor, and Shaele) showed low nucleotide and haplotype diversities compared to their counterparts from Saudi Arabia (Supplementary Figure 2).

### Haplotype Frequencies and Relationships

The already established haplotype nomenclature was slightly modified to correct discrepancies in a published study (Almathen et al., 2016). The first discrepancy was that identical sequences were assigned different names [e.g., haplotype B59 (JX946241) is identical to B73 (KF719287), B60 (JX946242) is identical to B74 (KF719288), and A65 (JX946240) is identical to A75 (KF719289)]. The second discrepancy was that two haplotypes were named after haplogroup B while belonging to haplogroup A (B69 and B76). Furthermore, nine new haplotypes were discovered in the current study (Supplementary Figure 3).

**FIGURE 2:**
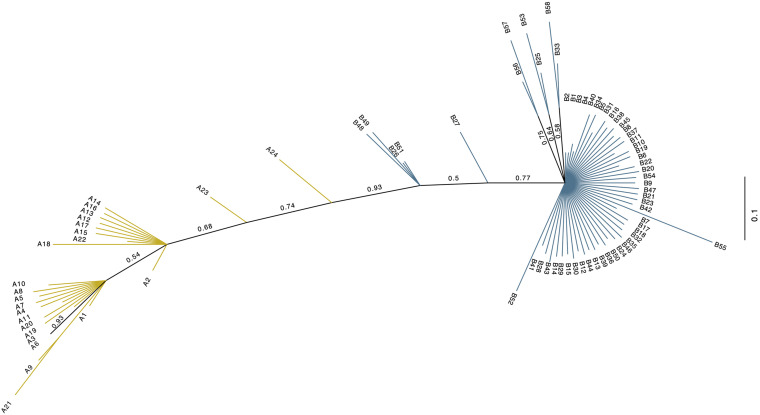
Unrooted phylogenetic tree of the 82 dromedary camel haplotypes. The new haplotype naming system based on this study. Values above branches are posterior probabilities (PP). Blue branch lines indicate haplogroup B sequences with posterior probability values = 1, while the yellow line designate haplogroup A sequences with a posterior probability = 1. Branches colored in black have *PP* values < 1.

**FIGURE 3:**
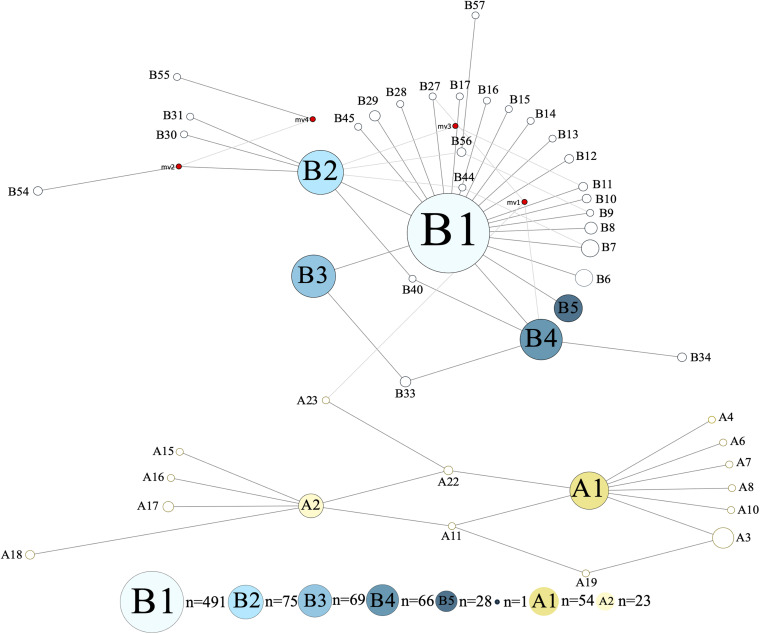
Median Joining Network of 867 bp camel mtDNA sequences. Singletons were excluded resulting in 47 haplotypes. Red circles represent median vectors. Colors are based on haplotype identity and frequency.

The investigated dromedary camel samples (*n* = 972 sequences) were represented by two haplogroups (A and B). Most haplotypes were classified under haplogroup B, which together contained 58 unique haplotypes (B1–B58), while haplogroup A contained 24 haplotypes (A1–A24) (Figure 2). Haplotype sequences (only parsimony informative sites) along with their accession numbers, and old and new names are listed in Supplementary Table 5. Among the 82 identified haplotypes, seven exhibited high frequency and were found in 806 dromedary camels (82.9%) and thus referred to as major haplotypes (B–B5 and A1–A2) (Supplementary Figure 4). A haplotypes exhibited the A allele at nucleotide position 375 (Mt reference position nt 15495) while B haplotypes showed the G allele. A Median Joining Network of the dromedary camel sequence haplotypes displayed the two haplogroups (A and B) connected to each other through a median vector (i.e., unsampled haplotype) (Supplementary Figure 4). Both haplogroups included a mixture of haplotypes of different frequencies in the analyzed populations. The interrelationships between mitochondrial haplotypes are illustrated in Figure 3.

When only D-loop (control region) was analyzed, 27 polymorphic sites were identified, which assigned the sequences to 58 haplotypes. Despite the decreased number of identified haplotypes compared to the analysis of the entire sequenced region, the proportions of haplotypes remained generally the same across dromedary camel-types and countries except for the low-frequency haplotypes (Supplementary Figure 5). Dromedary populations of some countries displayed a slight reduction in the proportions of A haplotypes (e.g., Egypt and Iran) (Supplementary Figure 5). Since the difference was minor, the entire region was used for all subsequent analyses, both to capture the maximum variation possible and to be consistent with previous studies (Almathen et al., 2016).

### Haplotypes in Relation to Population Names and Geography

**FIGURE 4:**
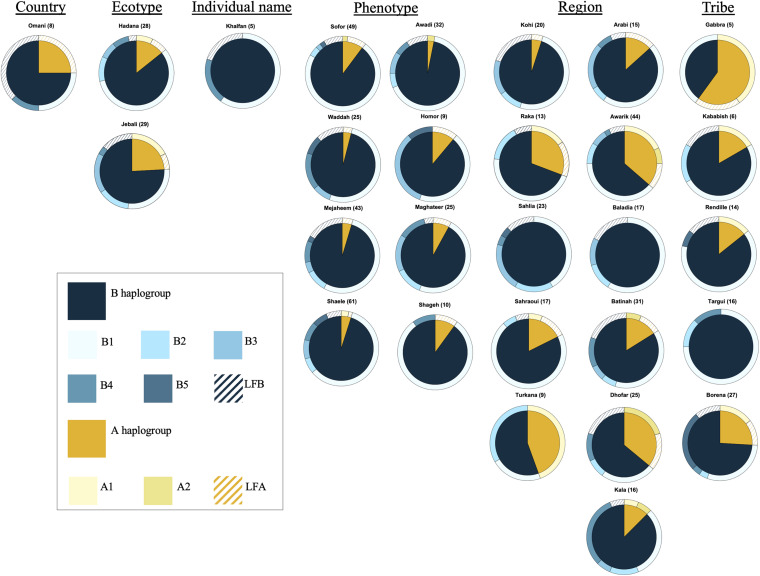
Frequencies of haplotypes of different camel-types grouped according to their naming criteria. Camel-types represented by <5 individuals were not plotted. Camel-types with unknown naming system are not shown.

The analysis of the haplotype variation of dromedary camel-types considering their names revealed that those named based on phenotype (including Mezayen types) were almost homogenous with reduced observed diversity compared to other naming systems (e.g., named after a geographic region). The A haplotypes were mainly represented with low frequency haplotypes, while the predominant B haplotype was B1 (Figure 4). The highest haplotype variation was observed within dromedary camel-types named after regions (different countries and continents). Camel-types named after tribal affiliation showed variation mainly in haplogroup A, but these types were represented by small sample sizes (Figure 4).

All haplotypes were observed across all geographic regions, but A haplotypes were slightly overrepresented in the region of the Horn of Africa (e.g., Somalia, Ethiopia, and Kenya). Haplogroup B was predominant in Asian countries and dromedaries from India and Yemen showed no A haplotypes and Pakistani camels had only ∼8% A haplotypes (Supplementary Figure 6). The haplotype frequency pattern in individual dromedary camel-types from each country was further investigated (Supplementary Figure 7). Raidi and Kohi, which are both Pakistani camel-types displayed differences in haplotype pattern especially in B haplotypes where the Kohi type has a comparatively higher number of low frequency haplotypes. Similarly, the Algerian camel-types, Targui and Sahraoui, showed similar patterns; the Targui type had no A haplotypes and no low frequency B haplotypes (Supplementary Figure 7). Dromedary camel-types located in the southern regions of Africa and in the Arabian Peninsula displayed increased proportions of A haplotypes. For instance, Omani, Batinah, and Dhofar types had ≤40% A haplotypes as well as the Awarik of Saudi Arabia. Among the dromedary camel-types that lacked haplogroup A were the Baladia (Sudan), Ja (Niger), Targui (Algeria), and the Sahlia (from Saudi Arabia) (Supplementary Figure 7).

### Pairwise Relationships Among Dromedary Camel-Types

**FIGURE 5:**
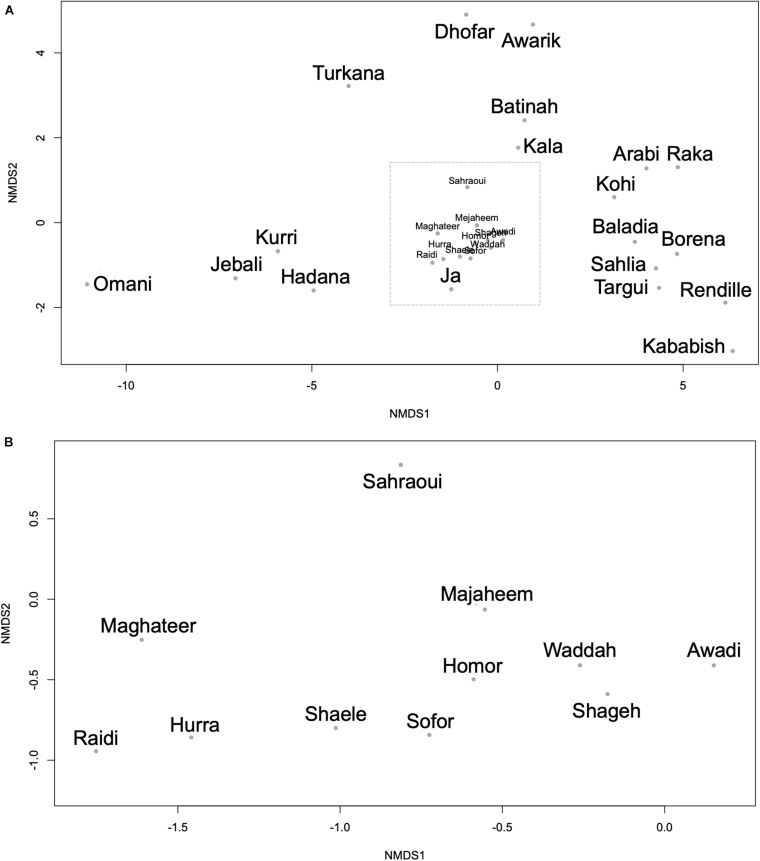
Non-metric multidimensional scaling plot constructed using the *F*_st_ values between each pair of the 30 camel-types. **(A)** All camel-types. **(B)** Expanded view of the camel-types enclosed in the dashed box in panel **(A)**. Each dot represents a camel-type.

Using the genetic difference index (*F*_st_), several dromedary camel-types exhibited significant genetic differences (Supplementary Table 6). The Raka (Syrian camel-type) and the Baladia (Sudanese camel-type) types displayed significant genetic differences from the majority of the other camel-types, whereas the Borena (Ethiopian camel-type) type exhibited no genetic differences from all other camel-types. Among camel-types from Kuwait and Saudi Arabia, Sahlia, Hadana, Awadi, and Majaheem appeared genetically different from all other local and distant dromedary camel-types (Supplementary Table 6). The Sahlia type was significantly difference from most dromedary camel-types, even with other camel-types located within the same country and was the only type of Saudi Arabia that was statistically different from the Kenyan and the Nigerian camel-types (Supplementary Table 6). Similarly, the Pakistani camel-types, Raidi and Kohi, were also significantly difference from one another despite residing in the same geographic locality. Camel-types within Africa such as Kababish and Baladia were genetically distant from one another, while the Kababish, Kala and the Ja types did not show significant genetic differences (Supplementary Table 6). Dromedary camel-type differentiation was inspected visually using an NMDS plot (Figure 5). A distinct cluster of African dromedary camel-types was observed with only the Kurri and the Turkana camel-types separated as distinct groups. Omani, Batinah, and Dhofar types were distant from each other, despite being in the same country (Oman) (Figure 5A). Awarik and Hadana were both genetically distant from other dromedary camel-types of Saudi Arabia. Mezayen camel-types clustered together, with the Maghateer being most distant from the centroid of this group (Figure 5B).

The relationships between dromedary camel-types of the same country were visualized using separate NMDS plots. This analysis revealed that the Kuwaiti dromedary camel-types were homogenous as well as the shared camel-types between Kuwait and Saudi Arabia (i.e., Majaheem, Sofor, and Shaele) (Supplementary Figure 8). However, collectively dromedary camel-types from Saudi Arabia were largely heterogenous and varied greatly from each other. The Omani, Pakistani, and Algerian camel-types were distant from each other within their respective countries. Although camel-types from Sudan clustered together, they showed more variation between one another than camel-types from Kuwait for example. Camel-types from Kenya were very distant from each other on the NMDS plot compared to Sudanese camel-types (Supplementary Figure 8). When AMOVA test was run on the whole data set, it was found that 91.93% of genetic variation was distributed within dromedary camel-types and only 8.07% was distributed between types with *F*_*st*_ value of 0.081 and *p*-value < 0.001 (percentages were for the obtained covariates).

A preliminary result showed that dromedary camel haplotypes were distinct from other camelids including guanacos, vicuñas, and Bactrian camels, and formed a monophyletic group including archeological samples (Supplementary Figure 9). Deep clades were supported by bootstrap values greater than 50. However, most of the clade showed low bootstrap values that might be a result of relatively short sequences used. Additionally, the dromedary haplotypes were the most derived sequences. Four out of the five used archeological samples clustered with the B haplotypes. The A haplotypes were ancestral to the B haplotypes, despite their low frequency among the sampled dromedary camel-types. Wild Bactrian camels shared the closest ancestry to the dromedary camel haplotypes (Supplementary Figure 9).

### Mezayen Camel-Types

**FIGURE 6:**
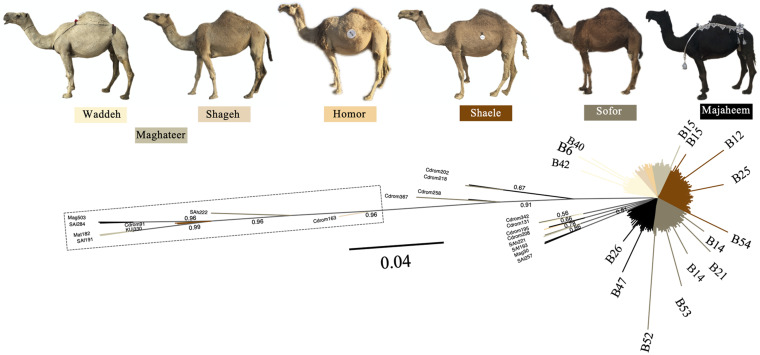
Unrooted phylogenetic tree of Mezayen camel-type haplotypes. Values above branches correspond to the posterior probability values. Posterior probability values = 1 were omitted for clarity. The dashed box contains haplotype A sequences. Non-major (low frequency) B haplotypes were illustrated at the tip of the branches. Photos are taken by HA.

The Mezayen camel-types were predominantly represented by the B haplogroup and more specifically B1 haplotypes (Supplementary Figure 10A). No clear distinction in haplotype frequencies was observed between the two subgroups of Mezayen camel-types [i.e., Majaheem (black colored) and Malaween (multicolored)]. The same general pattern was found in the proportions of the A haplotypes (Supplementary Figures 10B,C). Most Mezayen samples exhibited B haplotypes and only ∼4% were of A haplotypes (a total of eight camels had A haplotypes, of which only two samples had A1 and only one had the A2 haplotype) (Figure 6 and Supplementary Figure 11). Few samples belonged to A haplotypes, and those included Shaele, Shageh, Sofor, Majaheem, and Maghateer camel-types. Among these haplotype A dromedary camels, only two samples were from Kuwait, and the rest were from Saudi Arabia (Figure 6). An examination of the genetic difference (*F*_st_) among the Mezayen camel-types indicated a significant difference between the Majaheem camels to each of the Shaele, Homor, Shageh, and Maghateer camels (Supplementary Table 7). Sofor camels, on the other hand, was like all other Malaween camels except for Homor camels.

## Discussion

Different dromedary camel-types have been named, yet little or no documentation can be found about their breed status (Arman, 2007; Lynghaug, 2009; Porter et al., 2016; Alhaddad and Alhajeri, 2019). Several camel-type naming systems were previously described such as those based on ecotype (e.g., hill and riverine), country (e.g., Omani and Sudani), region of origin (e.g., Raka and Turkana), tribal affiliation (e.g., Kenani and Borena), and phenotype (e.g., Waddah and Shaele) (Leese, 1927; Mburu et al., 2003; Mehta et al., 2006; Ishag et al., 2010; Mahrous et al., 2011; Porter et al., 2016; Saad et al., 2017). Dromedary camel-type names currently in use in the literature exceed 200; all of which lacked registries and standard breeding criteria (Porter et al., 2016). Therefore, it is a necessary to investigate the relationships between dromedary camel-types and assess their population structure (Alaskar et al., 2021). The population structure and breed status examination of dromedary camel-types have recently been investigated using several STR markers (AlAskar et al., 2020). Analyzing mtDNA polymorphism is another way to evaluate the genetic relationships between dromedary camel-types (Hutchison Iii et al., 1974; Atig et al., 2009; Ahmed et al., 2016; Almathen et al., 2016; Saad et al., 2017). A general overview of mtDNA sequences revealed that across the sequenced region, 48 recognized variable sites were mostly transitions (Jukes, 1987; Almathen et al., 2016). As expected, most of the variable sites were found in the D-loop region, as it has lower selection pressure compared to the coding regions (McMillan and Palumbi, 1997).

### Overall Genetic Diversity

Both the nucleotide and the haplotype diversity indices suggested low differences between the dromedary camel-types, as indicated by the very low nucleotide diversity (0.00026) and the moderate to high haplotype diversity (0.725). These diversity indices might indicate a much lower selection pressure on the camels when compared to other livestock that are more intensively bred for a specified function.

This study updated the existing haplotype naming system to correct the inconsistencies previously reported (Almathen et al., 2016). Two haplogroups (A and B) were identified denoting two different maternal lineages. The current study identified a total of 82 haplotypes in dromedary camels. This is relatively high when compared to Bactrians, llamas, and vicuñas, which only had 15, 17, and 57 haplotypes, respectively (Ming et al., 2017; Casey et al., 2018; González et al., 2019). However, these numbers might be correlated with the overall population size of the different camelid species, since dromedaries have a much higher population size than these other species (FAO, 2013, 2017). Most previous studies that investigated maternal lines of species or breeds used the D-loop region for its high molecular variability (Kavar et al., 1999; Doosti and Dehkordi, 2011; Kawabe et al., 2014). This study used additional mitochondrial regions (coding genes) since these additional regions did not alter the resulting haplotype frequencies (Supplementary Figure 5).

### Relationship Between mtDNA Haplotypes and Dromedary Camel-Type Naming Criteria

Dromedary camel-types are named based on: names of individual camels, country names, ecotypes, phenotype, regions, tribal affiliation, and other criteria (Porter et al., 2016; Alaskar et al., 2021). This study aimed to test the hypothesis that dromedary camel-types would display haplotype variability depending on their names (AlAskar et al., 2020, Alaskar et al., 2021). Dromedary camel-types named after the name of an individual camel most likely represent lineages. An example of this camel-type is the Khalfan, which represents a racing camel-type in the UAE (Porter et al., 2016). A single haplotype was observed for the five analyzed Khalfan camels, which supports the hypothesis that types named after individuals are generally a homogenous group and represent a lineage. Dromedary camel-types named after ecotypes (e.g., Sahlia means beach camels, Hadana meaning hill camels, and Jebali meaning mountain camels) exhibited variation both in the A and the B haplotypes, indicating that these camel-types are mixtures of dromedaries of multiple origins or camel-types that experience recurrent introduction of genetically distant dromedaries. Also, this variability in haplotypes within ecotypic camel-types shows that there is no genetic uniqueness among individuals (i.e., signs of a breed). The Sahlia camel-type (meaning beach camels) differed in the haplotype frequency pattern from the other dromedary camel-types especially of the same country, mostly due to the lack of A haplotypes. This camel-type is located near the coast of the Red Sea, and is characterized by being short, and having round humps, narrow feet, and a short, thick neck (Porter et al., 2016). Therefore, it is likely that Sahlia camel-types possess distinct adaptive traits, which are suited for the high humidity of the coastal habitat. These adaptive traits and the inability of other types to flourish in the same habitat results in Sahlia’s reduced gene flow with neighboring dromedary camel-types.

Although the Omani camel-type is broadly named after a country, it was genetically different from almost all other camel-types, which suggests a genetically homogeneous group that approaches the status of a true breed. Dromedary camel-types named after tribes (e.g., Rendille and Targui) and geographic regions (e.g., Awarik, Baladia, and Batinah) generally display high variability in haplotype composition, which is indicative of admixture amongst its individuals with other populations. This is in accordance with previously reported measures of genetic variability reports (Mburu et al., 2003; Legesse et al., 2018). Dromedary camel-types named based on phenotype (e.g., Majaheem, Waddah, and Awadi) are usually selectively bred for distinctive phenotypes (e.g., coat color) (Almathen et al., 2016; Porter et al., 2016; Saad et al., 2017; Alhaddad and Alhajeri, 2019). These camels formed the most homogenous group based on haplotype; with B haplotypes being overrepresented in this group. This is in accordance with previously reported findings using microsatellite data (Mahmoud et al., 2019). The handful of individuals with A haplotypes within the Mezayen camel-types signify possible crossbreeding with distant camel-types.

### Dromedary Camel-Type Population Structure

Although no distinct phylogeographic structure was observed as previously reported (Almathen et al., 2016), A haplotypes were relatively of high frequency in African countries compared to Asian countries, except for Syria (Supplementary Figure 7). The low frequency of A haplotypes in Asian camels might be a result of periodic crossbreeding with African camels, since most African camel-types are of moderate to large body size (i.e., heavy pack) (Porter et al., 2016). Targui and Sahraoui are two camel-types of the Sahara (Oulad Belkhir et al., 2013) and despite occupying the same geographical locations, exhibited noticeable differences both in haplotype identity and frequency, which might be attributable to their phenotypic differences that limits intentional interbreeding (Oulad Belkhir et al., 2013). The Ja camel-type of Niger had no A haplotypes, which is unique among the dromedary camel-types of this country (Kala and Kurri). However, no genetic distinction was found when dromedary camel-types of Niger were analyzed using STR (Abdussamad et al., 2015; AlAskar et al., 2020).

Analysis of Molecular Variance results imply little or no clear sub-structuring both in relation to the dromedary camel-types and their geographical distributions. Most of the mtDNA variation was observed within the studied camel-type samples while 8% of the observed variation was among the 37 studied camel-types, which might be due to a shared origin of the two haplogroups and the continuous gene flow amongst the various dromedary camel-types. Pairwise *F*_st_ values revealed that some dromedary camel-types of the same geographical location (i.e., country) are genetically distant from one another (see Saudi Arabia, and Oman, etc.). This can be attributed to differences between camel owners, in selected camel qualities, and/or breeding systems (Al-Hazmi et al., 1994; Abdallah and Faye, 2012, 2013).

### Mezayen Dromedary Camel-Types

In general, a low frequency of A haplotypes was found in the six Mezayen camel-types. This low frequency suggests little crossbreeding with non-Mezayen dromedary camels. The mitochondrial relationships within the six Mezayen camel-types indicate that: (1) Majaheem camels are different from the rest of the Mezayen camel-types (i.e., Malaween), (2) Maghateer camels, which is a name given to different camel groups depending on their location and tribal ownership, appears to be more diverse than all other Mezayen types, and (3) Malaween camel-types are generally similar. These findings were supported by a study on dromedary camel torso using geometric morphometric methods (Alhajeri et al., 2019). The uniqueness of Majaheem camels, and its separation from the other Mezayen types agrees with the phenotypic differences that may prevent interbreeding and gene flow (Alhaddad and Alhajeri, 2019). The observed diversity of Maghateer camels may be related to the fact that certain camel breeders define this dromedary camel-type as a mixture of Waddah and Shageh camels or even all colored Mezayen camel-types (Alhaddad and Alhajeri, 2019).

## Conclusion

Dromedary camel mitochondrial haplotypes were more distinct than mitochondrial haplotypes in other camelids, and haplogroup A may represent the ancestral form of the more abundant B haplogroup. Little genetic difference can be observed between dromedary camel-types. The observed geographic distribution of the mitochondrial haplotypes could be due to the physical separation of the dromedary camel-types. Also, an introgression event could have helped to introduce the A haplotypes into the Asian camel-types. Mezayen camel-types most probably represent the true breeds of the Arabian Peninsula as they exhibited homogenic haplotype mixture as well as having a set of well-identified phenotypic traits as selection criteria. The investigation of camel mtDNA is probably not sufficient to fully explain the relationships between dromedary camel-types and identify true breeds. Nuclear genome markers such as SNPs, STR, or even whole genome sequencing should be used for more comprehensive conclusions to be reached.

## Data Availability Statement

The datasets presented in this study can be found in online repositories. The names of the repository/repositories and accession number(s) can be found below: https://www.ncbi.nlm.nih.gov/genbank/, MT164347 – MT164465.

## Author Contributions

RA and HA designed the experiments. HA, BA, and FA collected the samples. RA performed the experiments and analyzed the data. RA, HA, and BA wrote the manuscript. All authors contributed to the article and approved the submitted version.

## Conflict of Interest

The authors declare that the research was conducted in the absence of any commercial or financial relationships that could be construed as a potential conflict of interest.

## Publisher’s Note

All claims expressed in this article are solely those of the authors and do not necessarily represent those of their affiliated organizations, or those of the publisher, the editors and the reviewers. Any product that may be evaluated in this article, or claim that may be made by its manufacturer, is not guaranteed or endorsed by the publisher.
