# Supplementary material for: Mitochondrial Sequence Variation, Haplotype Diversity, and Relationships Among Dromedary Camel-Types
Source: Front Genet. 2021 Aug 30;12:723964. doi: 10.3389/fgene.2021.723964 (PMC8435798; doi:10.3389/fgene.2021.723964)
Supplement: Supplementary file 1 [file Data_Sheet_1.PDF]

Mitochondrial Sequence Variation, Haplotype Diversity, and Relationships Among Dromedary Camel-types

Randa Alaqeely[1], Bader H. Alhajeri[1], Faisal Almathen[2,3], and Hasan Alhaddad[1*]

[1]Department of Biological Sciences, Kuwait University, Safat, 13060, Kuwait

[2] Department of Veterinary Public Health and Animal Husbandry, College of Veterinary Medicine, King Faisal University, Al-Hasa, Saudi Arabia

[3]The Camel Research Center, King Faisal University, Al-Hasa, Saudi Arabia

[*]Corresponding author: hassan.alhaddad@ku.edu.kw

Supplementary material

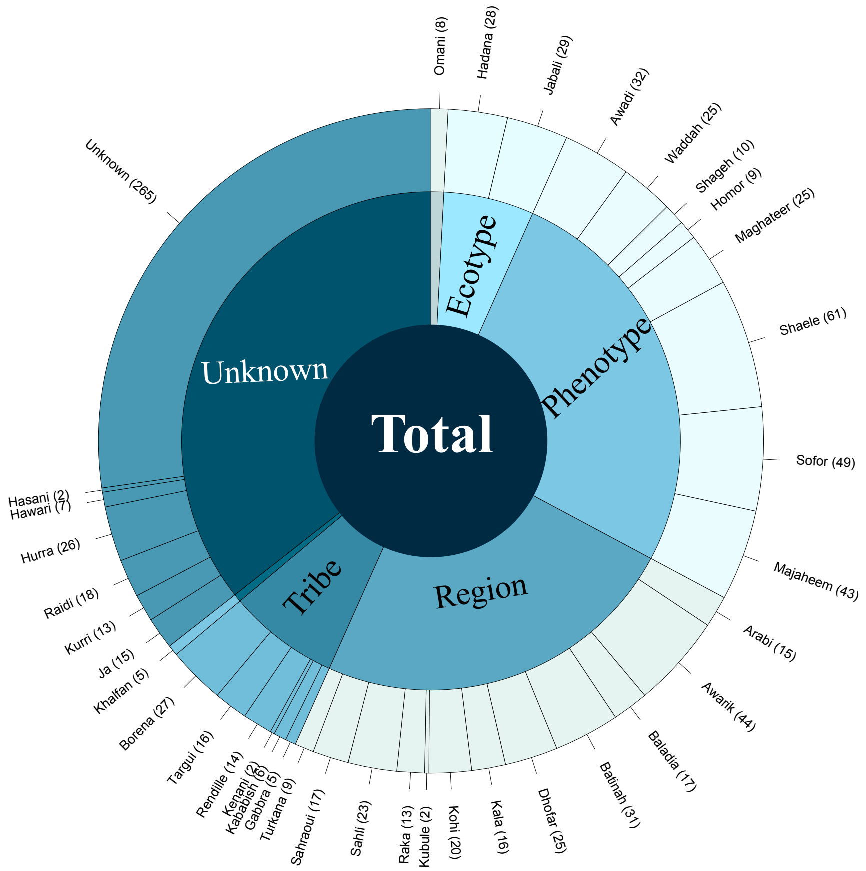

Figure S1: Numbers of the total mtDNA sequences (n=972) and their corresponding camel-type designation used in this study. The outer circle represents the number of sequences available for each camel-type whereas the middle (inner) circle represents the likely origin of the camel-type's name. Note: the Omani camel-type is named after country, while Khalfan is the name of an individual camel. Two camel-types are named after an ecotype (n=57), eight named after their phenotype (n=254), twelve named after a region (n=232), six named after tribal affiliation (n=70), and six had unknown naming criteria, in addition to unknown camel-types (n=346).

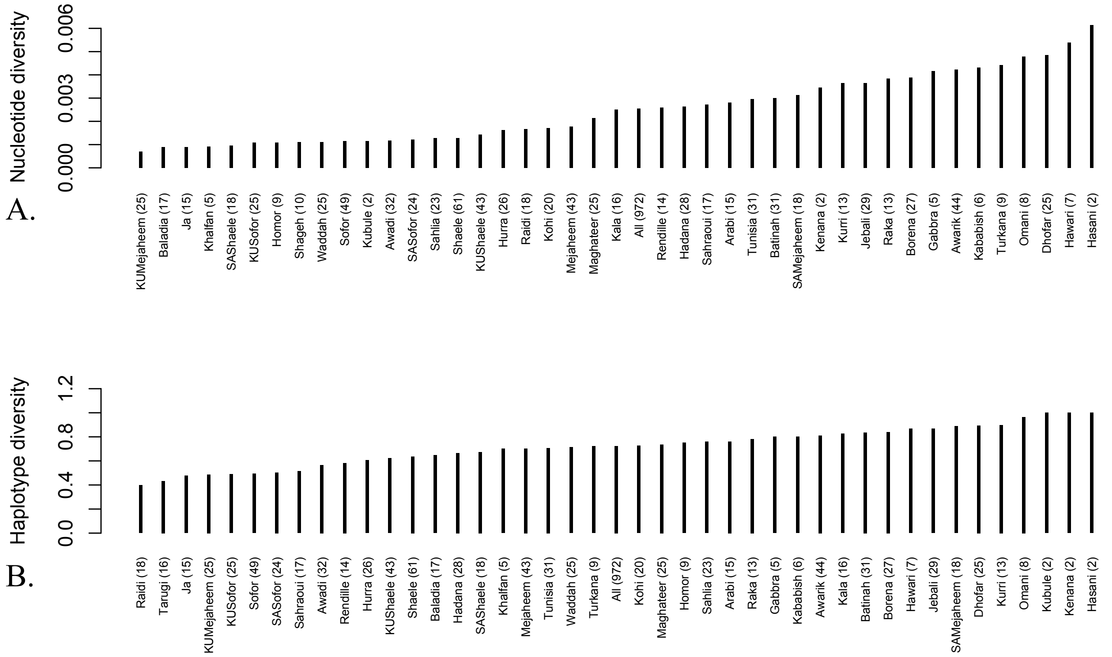

Figure S2: Nucleotide and haplotype diversities for investigated dromedary camel-types. A. nucleotide diversity. B. haplotype diversity. Unknown camel-types were excluded from this plot. Camel-types are represented in ascending order. Numbers between parenthesis indicate sample size.

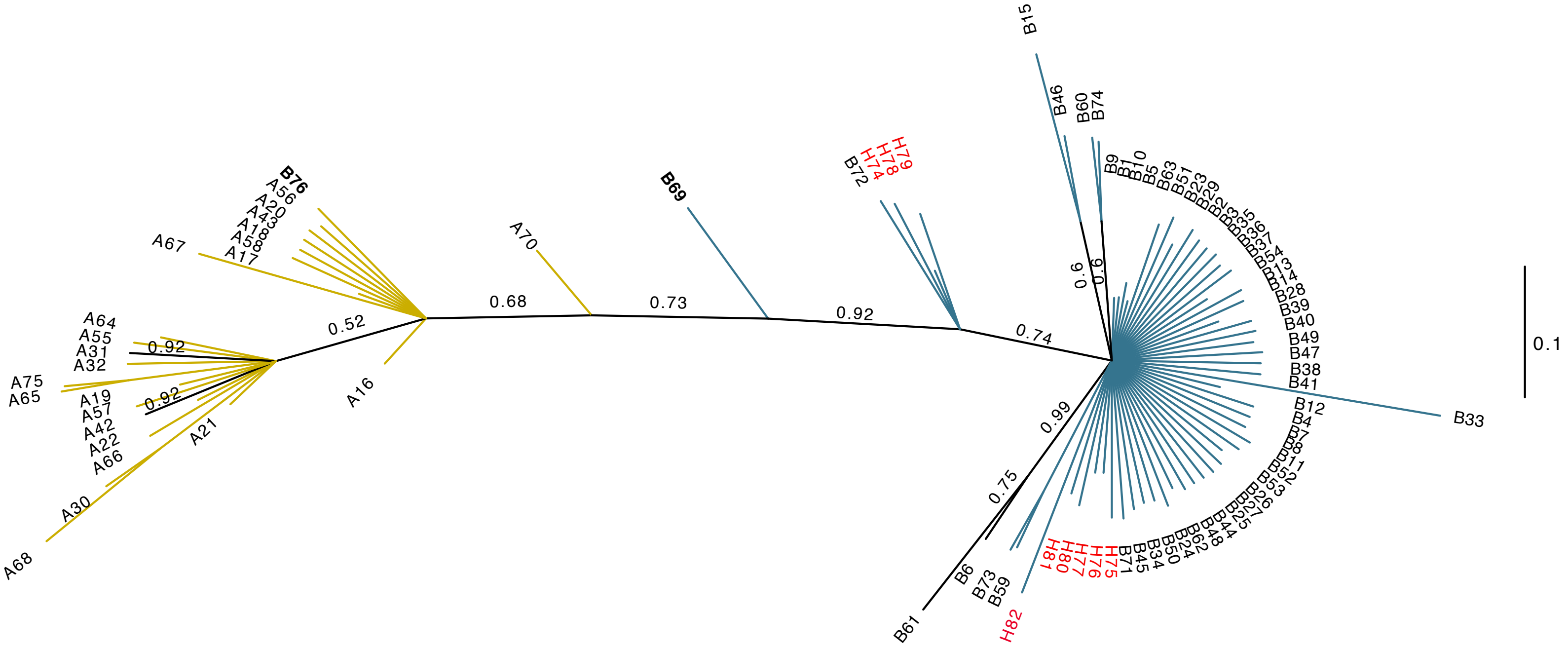

Figure S3: Unrooted phylogenetic tree of the 82 dromedary camel haplotypes. Nomenclature of haplotypes is based on (Almathen *et al.* 2016). Values above branches are posterior probabilities (PP). Haplotypes in bold were mis-assigned, and those in red are the new haplotypes based on this study.

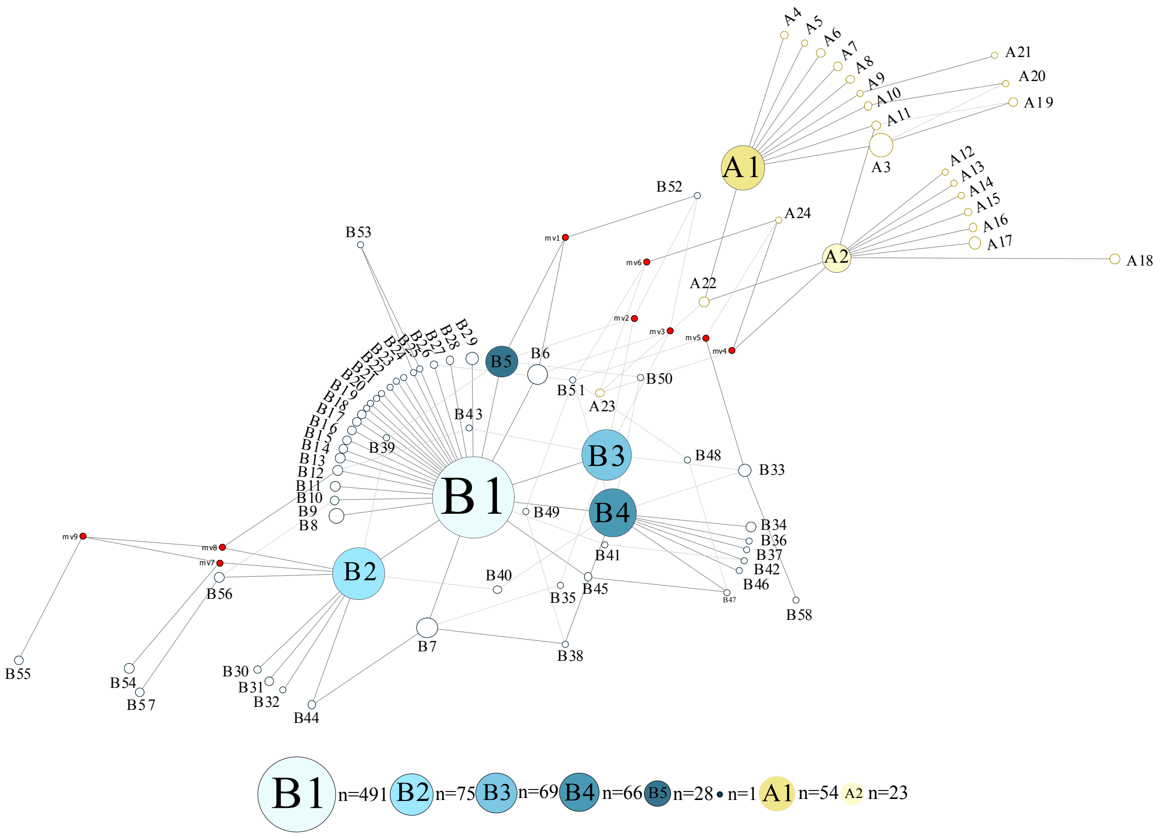

Figure S4: Camel mitochondrial haplotype network of 972 sequences. The Median Joining Network demonstrates the relationships between the 82 identified haplotypes that were grouped into two haplogroups (A and B). Red circles indicate median vectors that designate missing/unsampled haplotypes or extinct ancestral haplotypes. Networks are scaled (i.e. branch lengths are proportional to the number of mutations). Haplotypes are colored according to their identity and frequency. Unfilled circles correspond to low frequency haplotypes.

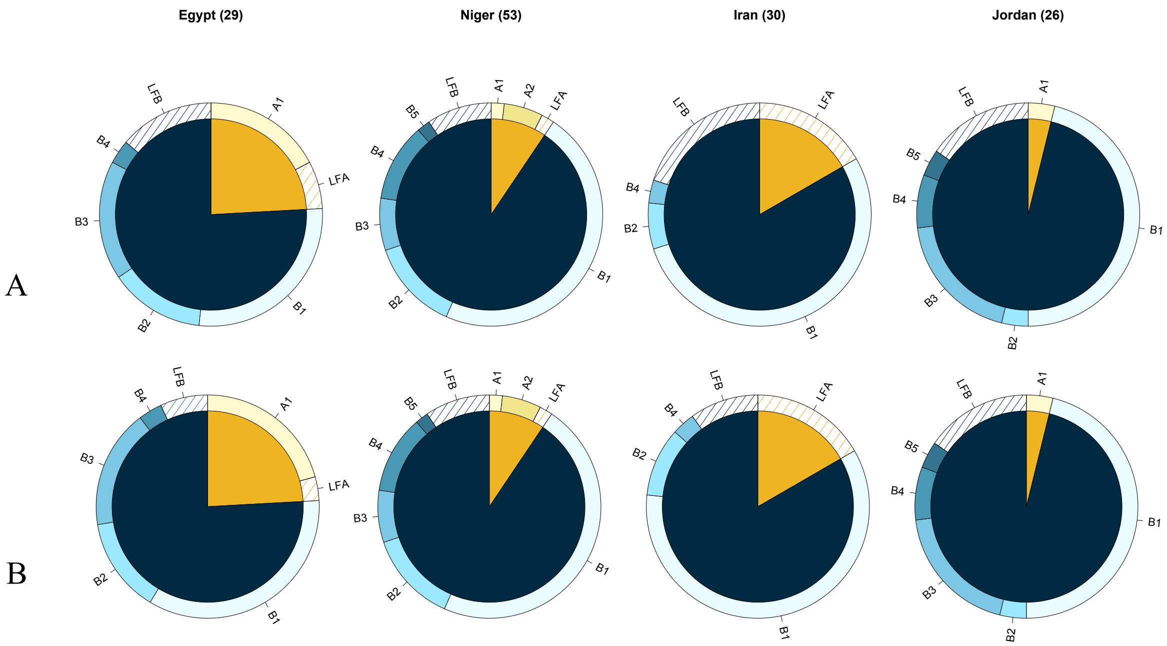

Figure S5: Proportions of haplotypes in camel populations from four countries using different mitochondrial DNA regions. A. Haplotype proportions assigned using the entire 867bp mitochondrial region. B. Haplotype proportions based on D-loop (552bp) only. LFA signifies low frequency A haplotypes, LFB indicates low frequency B haplotypes. Low frequency haplotypes were those with frequencies < 0.019. Numbers between parenthesis indicates sample size.

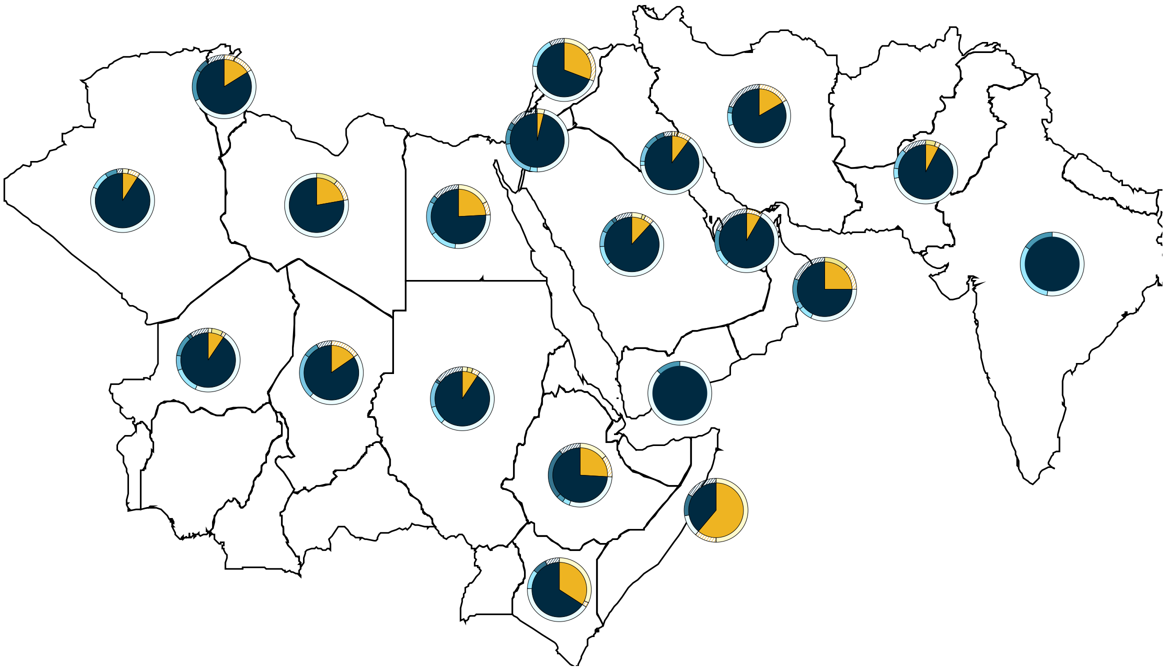

Figure S6: Proportions of camel haplotypes from different countries. Each country is represented by all sampled camel-types. Dashed lines indicate low frequency haplotypes, yellow color indicates for A haplotypes, and blue color indicates for B haplotypes.

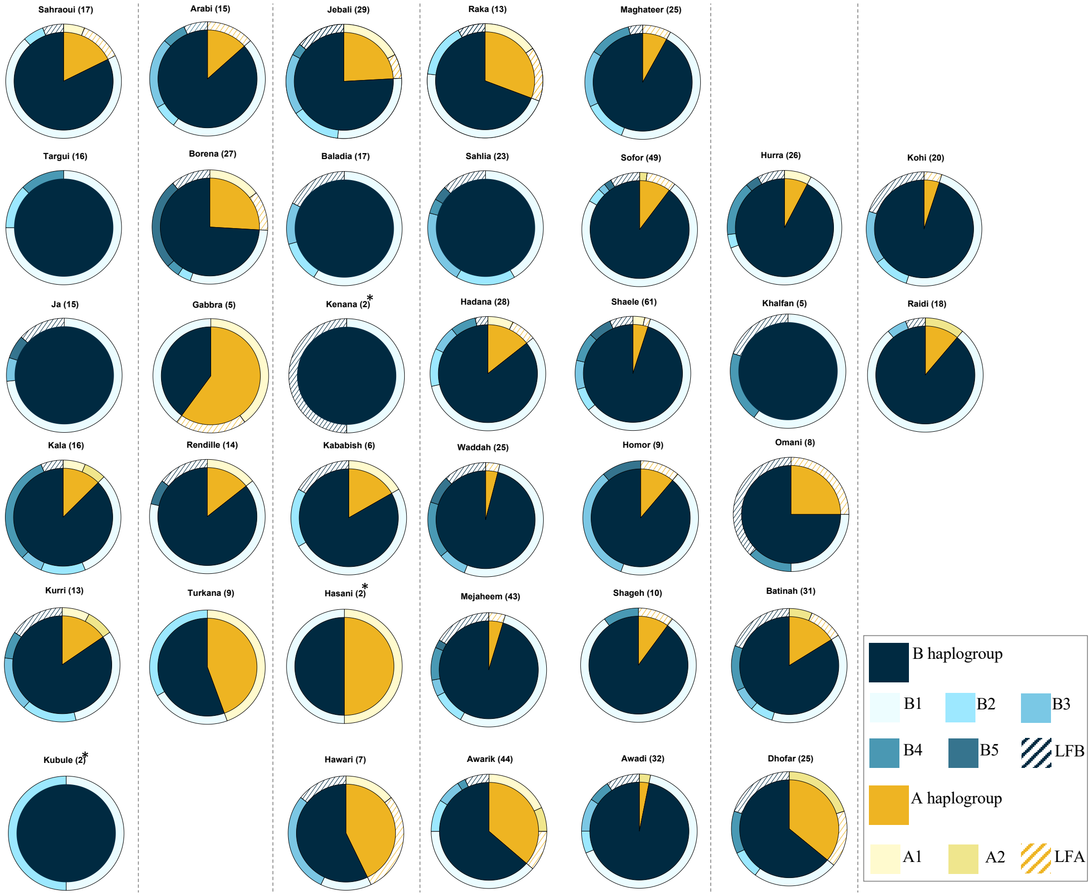

Figure S7: Proportions of haplotypes in 36 camel-types ordered based on their geographical location. Vertical dashed lines separate camel-types based longitudinal zones. Pie charts are roughly organized left to right (West to East) and top to bottom (North to South). Unknown camel-types were not included. Asterisks signifies low sample size. This representation is meant to give an overview of geographic relationships among camel-types, and the frequencies of the haplotypes.

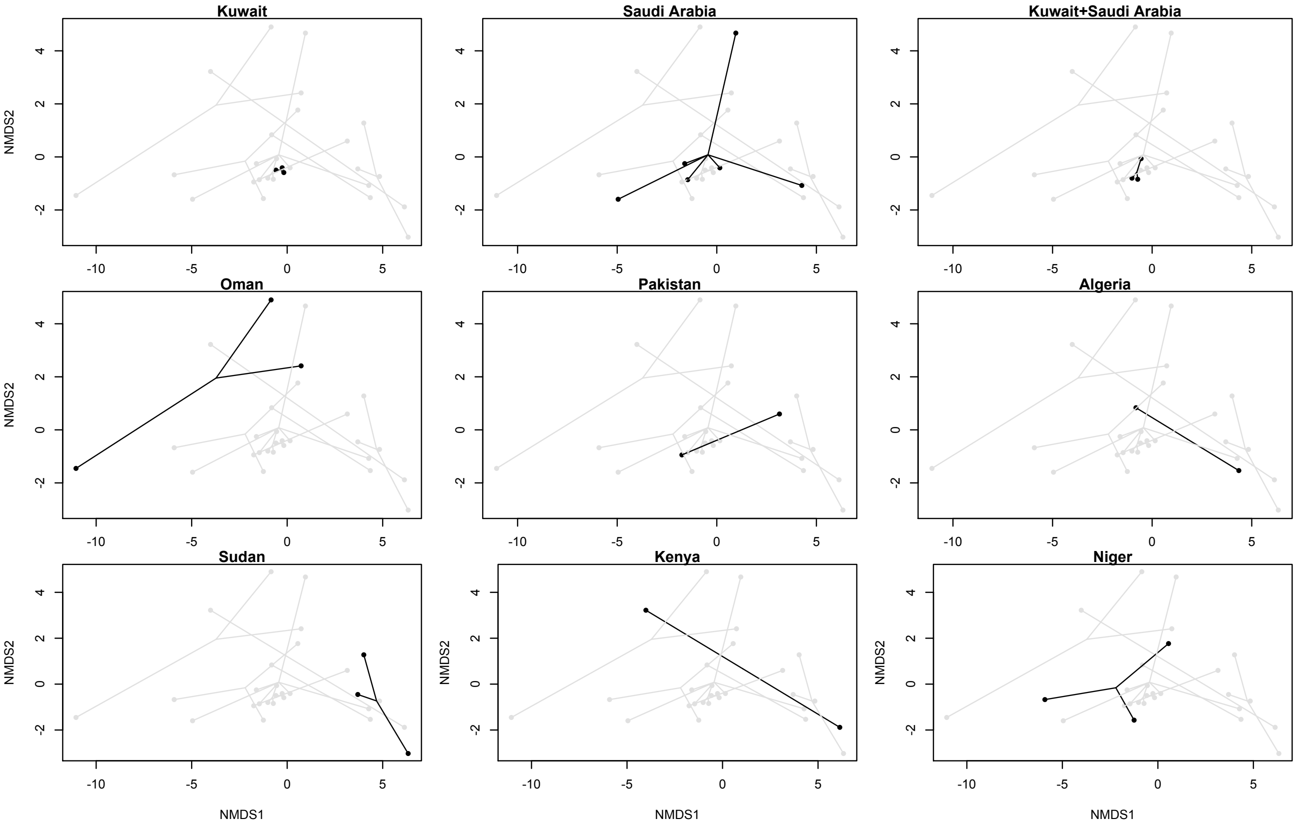

Figure S8: NMDS of the F_{st} values of the camel-types within countries. Only countries with represented by more than one camel-type were included. Black colored dots correspond to the camel-types of the indicated country, while gray dots denote other camel-types. Kuwait+ Saudi Arabia represent camel-types found in both Kuwait and Saudi Arabia, namely the Shaele, Majaheem, and the Sofor. The intersection points are centroids (averages) and the straight lines represent the genetic distance.

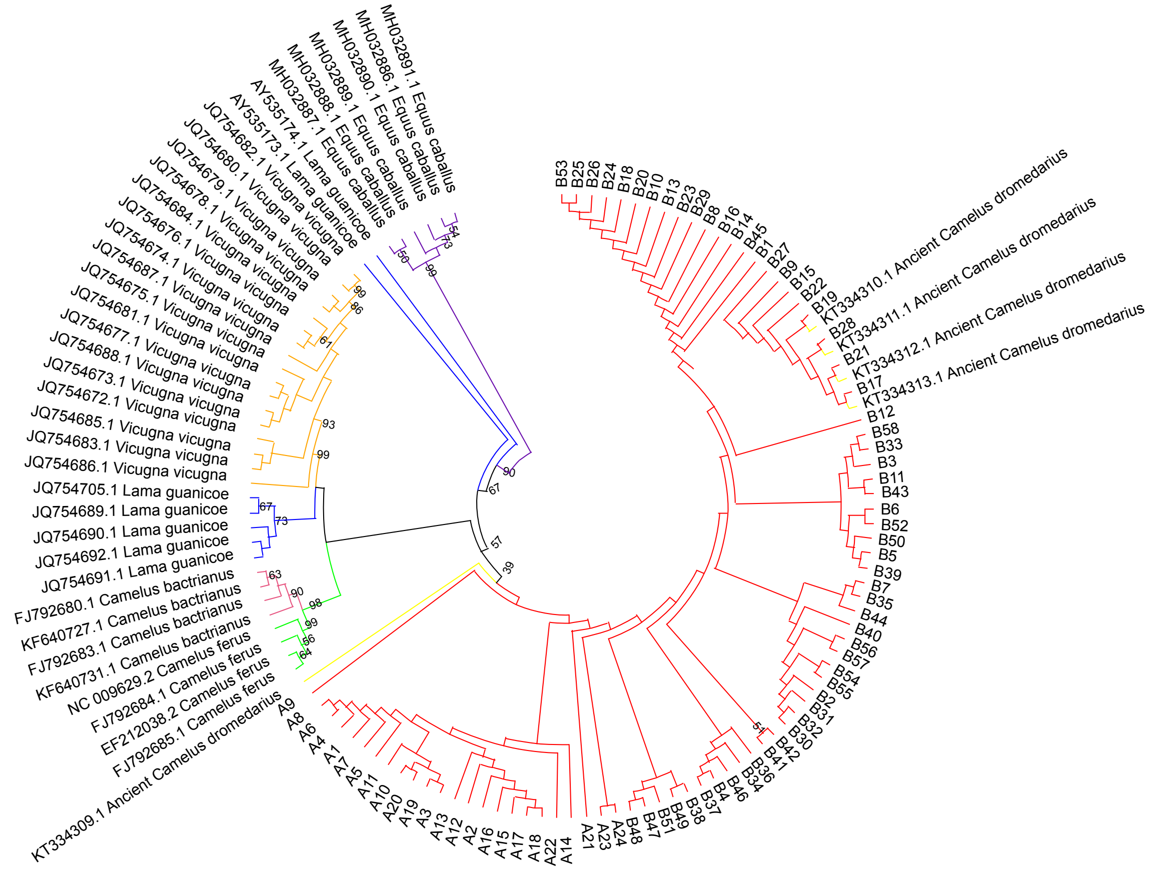

Figure S9: mtDNA-based Neighbor Joining Phylogenetic tree of five camelid species. Red lines represent mtDNA sequences of dromedary camels, Yellow lines represent Archeological (ancient) *Camelus dromedaries*, green lines represent wild Bactrian (*Camelus ferus*), Pink color represent Bactrian (*Camelus bactrianus*), blue lines represent guanaco (*Lama guanicoe*), orange color represent vicuña (*Vicugna vicugna*), and purple color represent horse samples (*Equus caballus*) as an outgroup. Node support was tested using 1000 bootstraps. Since sequences of each species differed in base pair length, dromedary sequences were cropped to match the length of other species. Numbers on the tree indicate bootstrap values and values that are less than 50 were not shown.

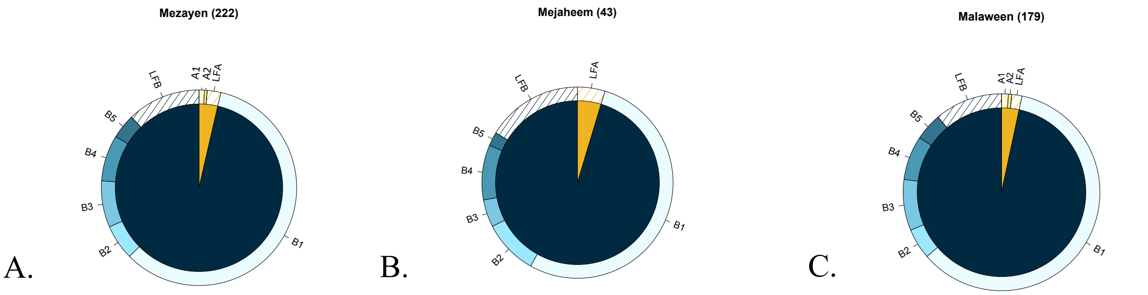

Figure S10: Mitochondrial haplotype frequencies in Mezayen camel-types. A. Haplotype proportions for all Mezayen camel-types. B. Haplotype proportions of Majaheem only. C. Haplotype proportions of Malaween only (Waddah, Shageh, Homor, Shaele, and Sofor).

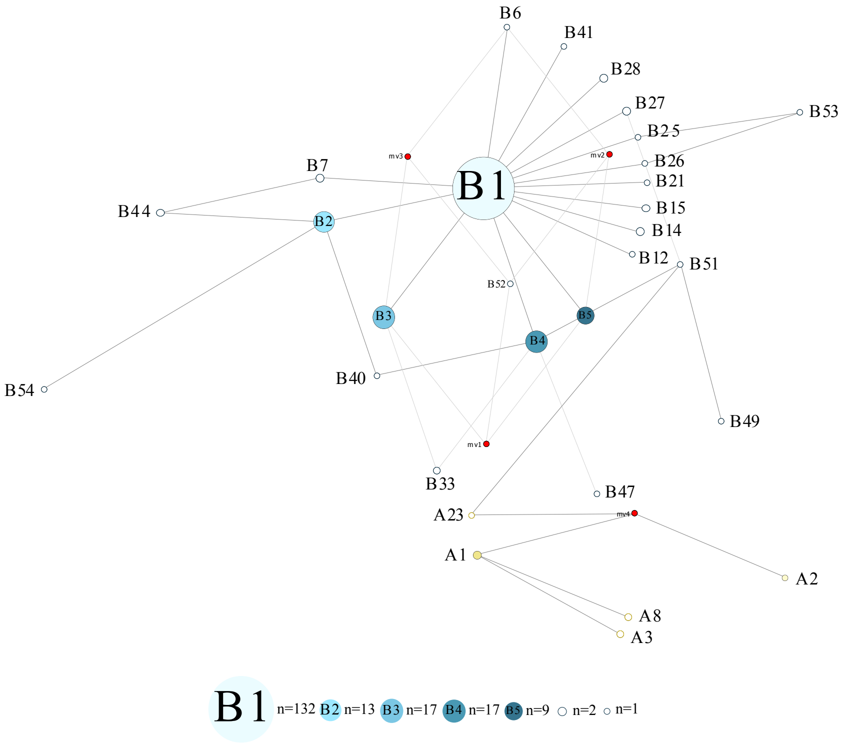

Figure S11: Haplotype relationships of Mezayen camel-types using the Median Joining Network analysis. Red circles signify median vectors (unsampled haplotypes).
